# Imidazoline As a Volatile Corrosion Inhibitor for Mitigation of Top- and Bottom-of-the-Line CO_2_ Corrosion in Carbon Steel Pipelines

**DOI:** 10.1021/acs.langmuir.3c03827

**Published:** 2024-05-30

**Authors:** Nattawut Yotapan, Nipaporn Sriplai, Sureeporn Ruengsangtongkul, Korakot Sombatmankhong

**Affiliations:** National Energy Technology Center (ENTEC), National Science and Technology Development Agency (NSTDA), 114 Thailand Science Park, Phahonyothin Road, Khlong Nueng, Khlong Luang, Pathum Thani 12120, Thailand

## Abstract

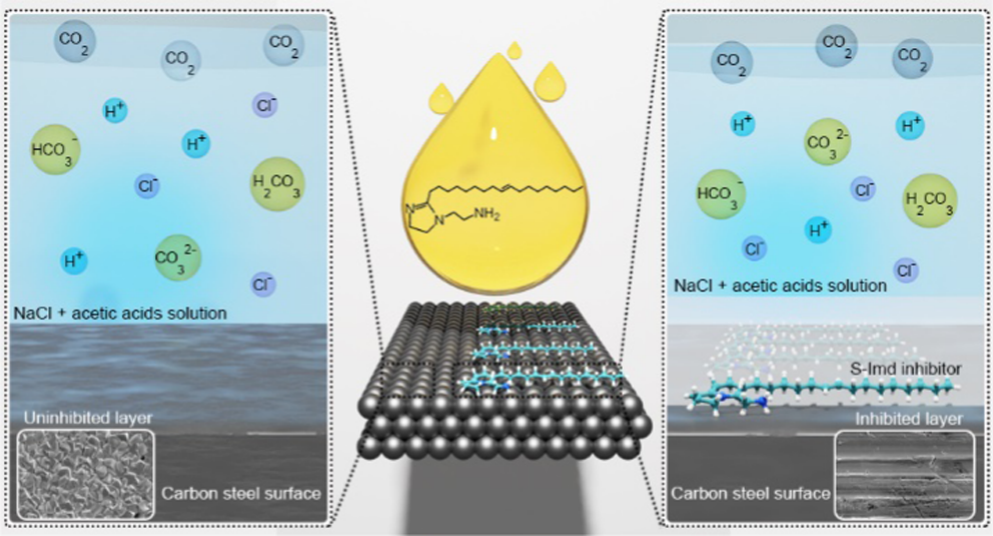

A fatty acid imidazoline-based inhibitor was synthesized via a facile solvent-free synthesis method between tall oil fatty acid (TOFA) and diethylenetriamine (DETA) under atmospheric conditions with a short reaction time. The as-synthesized imidazoline (S-Imd) acted as an effective inhibitor for reducing or preventing corrosion of carbon steel pipelines at both bottom of the line (BOL) and top of the line (TOL) positions under simulated conditions of a gas pipeline in a CO_2_-saturated environment. The inhibition efficacy was examined by both weight loss and electrochemical measurements, such as the electrochemical impedance spectrum (EIS), potentiodynamic polarization (PDP), and linear polarization resistance (LPR). The results revealed that the S-Imd, 2-(8-heptadecenyl)-2-imidazoline-1-ethanamin, at 300 ppm exhibited a superior inhibition efficiency of up to 91.6 and 89.9% for BOL and TOL corrosion tests, respectively. The surface morphology of the carbon steel test specimens was also examined using scanning electron microscopy (SEM), energy dispersive X-ray spectroscopy (EDAX), and contact angle analysis. It was found that the as-synthesized S-Imd acted as a mixed-type inhibitor that exhibited a decreased surface roughness and oxide layer on carbon steel surfaces. However, the water contact angle was found to increase, implying enhanced hydrophobicity of the surface. Adsorption of the imidazoline molecules on carbon steel surfaces followed the Langmuir adsorption isotherm. The present work provides very promising results in the synthesis and utilization of the studied imidazoline as a volatile corrosion inhibitor (VCI), especially for carbon steel pipelines in petroleum industries.

## Introduction

Top-of-the-line (TOL) and bottom-of-the-line (BOL) corrosions of pipelines are very important and challenging problems in the petroleum industry.^1,2^ In general, an internal pipeline system consists of two main parts, depending on the exposed areas and environmental conditions. TOL refers to the upper part of the pipeline at which moisture condensation occurs. BOL is in the lower part of the pipeline, which is in contact with a flowing liquid phase (condensate water and brine). Under wet gas pipeline conditions in the presence of CO_2_ gas and moisture, dissolved CO_2_ reacts with H_2_O, yielding H_2_CO_3_, which is then dissociated to H^+^, HCO_3_^–^, and CO_3_^2–^. This leads to a decline in the pH of condensed water situated on the upper part of the pipeline.^3,4^ TOL corrosion is therefore a significant problem in wet gas pipelines due to its critical location for the condensed water aggravated by different temperature gradients between the pipe wall and the external environment.^5^ This brings about serious corrosion of carbon steel pipelines in the presence of CO_2_.^1^ Although there are several approaches to protect metal surfaces and reduce CO_2_ corrosion from attack by diverse corrosive media, addition of corrosion inhibitors for internal pipeline protection is one of the most practical and cost-effective methods.^6,7^ Moreover, this approach can dramatically reduce corrosion rates (CRs), even at a low dose of 10 ppm or sometimes at higher levels of up to 5000 ppm, depending on the operating conditions and impurities.^6^

Protection using corrosion inhibitors is mainly based on modification of metal surfaces through adsorption of inhibitor molecules and subsequent formation of a protective blocking film layer.^3^ For the TOL area in the gas pipeline, high vapor pressure of corrosion inhibitors is needed (so-called VCI(s)) so that the inhibitor molecules can be vaporized and form a barrier layer with high passivation properties that prevents corrosion of carbon steel in the presence of CO_2_, O_2_, salt, acid vapors, and/or corrosive materials.^8^ In practice, the extraordinary properties of VCI molecules are their excellent inhibition efficacy for both TOL and BOL corrosions in pipelines. VCI molecules basically volatilize from a solid or a liquid into a gas/vapor phase and then are distributed throughout the top space of pipelines. This results in a protective molecular layer on the metal surfaces that prevents exposure to corrosive species, thereby minimizing corrosion. The VCIs are perfect for preventing rust in enclosed spaces or complex equipment because they can reach hidden and hard-to-reach areas unlike traditional inhibitors that need direct contact with the metal. VCIs are highly volatile in nature. They are available in various forms such as films, papers, sprays, and oils, which can protect a wide range of metallic materials including steel, aluminum, and copper. VCIs are also environmentally friendly, as they do not contain any toxic substances and can be easily disposed. However, VCIs are best effective for a uniform and clean metal surface. Otherwise, the adsorption of VCIs on a metal surface could not be uniform, and therefore, the corrosion rate is mainly governed by the propagation of defects such as the breakage of the VCI protection film.^9^ Heterocyclic organic compounds that contain multiple bonds and heteroatoms such as O, N, or S are excellent corrosion inhibitors because they could be adsorbed on the metal surface through these heteroatoms. The absorbance of such compounds on the metal surface blocks active sites and reduces the corrosion rate. However, the inhibitor’s effectiveness depends on the physical and chemical properties of the inhibitor structure due to the existence of specific functional groups, aromaticity, electronic density, corrosive solution, and the structure of the inhibitor. Recently, several studies have approved that many heterocyclic compounds such as imidazoline derivatives have a better inhibitory role on carbon steel in acidic medium.^10−16^

Furman and Kharshan^17^ suggested that the most effective corrosion inhibitors for oil and gas pipeline applications were the fatty acid imidazoline-type materials. Currently, long-chain imidazoline compounds are extensively developed for preventing or reducing the corrosion of carbon steel in oil and gas pipelines. They have excellent corrosion inhibition performance, low toxicity, ease of synthesis, and high efficacy for corrosion inhibition, along with environmental friendliness and high cost effectiveness in terms of corrosion inhibition.^18,19^ The chemical structure of imidazoline consists of three main groups, an imidazoline headgroup, a pendant group, and a hydrocarbon tail.^2,20^ An imidazoline ring with the presence of π-electron and nitrogen heteroatoms is rich in lone pair electrons that facilitate strong bonding of the molecule to steel surfaces.^20,21^ The pendant group or alkyl amine substituent of the imidazoline ring acts as an anchor that helps maintain its adsorption on steel surfaces^21^ and promotes its solubility in a mixture of oil and water in a gas pipeline. Moreover, the nonpolar long hydrocarbon chain, which is commonly known as the hydrophobic tail group in the imidazoline structure, formed as a hydrophobic layer on metal surfaces and thus hinders the interaction with water/oxygen and the corrosive environment.^22^

Imidazoline-based inhibitors were synthesized via a simple amidation reaction between natural fatty acids and various amines in a reaction that required over 5 h to form imidazoline rings.^23−26^ Additionally, some organic solvents such as toluene^23^ or xylene^24^ were also added to the synthesis reaction of imidazoline compounds. Alternatively, a microwave synthesis could greatly shorten the reaction time of imidazoline formation to a few minutes, but the production volume or scalability of such a process is limited.^27−29^ A facile synthesis process of imidazoline-based inhibitors by means of modulating reaction temperature and pressure was employed in the present work under solvent-free conditions to develop the most practical and cost-effective method for further commercialization. Basically, there are two steps used for the formation of imidazoline compounds: amidation and cyclization. Initially, the carboxylic acid group(s) in fatty acids reacts with the amine group(s) in polyamine compounds through an amidation reaction. Then, an intramolecular cyclization reaction yields the corresponding imidazoline ring. A variety of starting materials have been studied by several research groups.^30−32^ The most used fatty acids are stearic acid, palmitic acid, lauric acid, oleic acid, and some oils. Tall oil fatty acids (TOFAs) are common byproducts of coniferous wood processed in the pulping industry and are one of the low-cost raw materials for the synthesis of imidazoline-based inhibitors at an expected cost of ∼0.02–3 USD/m^3^. Examples of the polyamines are ethylenediamine (EDA), diethylenetriamine (DETA), aminoethylethanolamine (AEEA), triethylenetetramine (TETA), and tetraethylenepentamine (TEPA).^30^ From the literature,^33^ it is found that DETA is commonly used as an inhibitory organic polyamine compound for corrosion mitigation of steel-based materials in various acidic and brine solutions. It is an active primary aliphatic amine with five reactive hydrogen atoms that readily interact with the active sites of many compounds or molecules. Hence, the molecular architecture of branched macromolecules containing DETA polyamine and TOFA constituents significantly improves their inhibition properties against the aggressive attack of steel corrosion.

Ditama et al.^34^ synthesized imidazoline-based inhibitors via a reaction between methyl oleic fatty acid and *N*-(2-aminoethyl)-3-aminopropyltrimethoxysilane under an inert atmosphere with a reaction time of over 17 h. In the same way, imidazoline derivatives were prepared using a total reaction time of 13 h.^29^ Geng et al.^4^ prepared rosin imidazoline by adding TETA in a mixture of rosin and xylene solvents at 120 °C for 4 h and then increased the reaction temperature to 220 °C for 6 h. Moreover, synthesis of 2-[2-(7-isopropyl-1,4-dimethyl-9,10-octahydro-phenanthren-1-yl)-4,5-dihydro-1-yl]-ethylamino-methyl-phosphonicimidazole was performed by means of an amidation reaction (at 140 °C for 2 h) and a cyclization reaction (220 °C refluxed for 2 h) between DETA and dehydroabietic acid in xylene.^35^ Zheng et al.^18^ produced an imidazoline derivative (mercapto-oleic imidazoline) from a reaction between oleic imidazoline and mercaptopropionic acid under agitation and reflux for 4 h. Clearly, most studies on the conventional synthesis methods rely on multiple time-consuming reaction steps, tedious workup, and high-purity reagent-grade chemicals (e.g., single fatty acids or mixtures of well-defined fatty acids but not much on TOFAs). This restricts further implementation in large-scale production of highly efficient imidazoline-based inhibitors at a reasonable price. Technically, the low volatility of imidazoline compounds limits their use as VCIs.^36^ Poor water solubility of imidazoline is also a significant technological challenge that impacts its inhibition efficacy in real applications. It has been reported that solvents such as alcohols, ethers, mineral spirits, acetates, naphthenic distillates, and glycols can be used to enhance the naturally low vapor pressure and solubility of organic compounds.^17^ For example, chlorofluorocarbon (CFC)-113 (CCl_2_F-CClF_2_) was used as a solvent for formulating VCIs composed of amines, carboxylic acids, and/or triazoles due to its nontoxicity, rapid evaporation, high surface wettability, and capability of dry film formation.^37^ 1,1,1-Trichloroethane was developed as an alternative solvent due to the ozone depletion potential of the CFC solvent, which helped to improve corrosion inhibition performance. Aiad et al.^38^ suggested that addition of ethanol at 10% v/v can improve their solubility and also enhance the inhibition efficiency of single fatty acids from 36 to 75% in 1 M hydrochloric and sulfuric acid to enhance the solubility of imidazoline in corrosive media. Addition of solvent and/or other additives does not promote the adsorption mechanism of a corrosion inhibitor on metal surfaces or offer extra metal protection.^39^ However, it encourages compatibility with the environment, increases vapor pressure, and/or makes viable the active transport to the TOL area to be protected.

The evaluation and development of anticorrosive properties of imidazolines have been focused mainly on corrosion protection against BOL corrosion through both chemical (weight loss) and electrochemical measurements.^4,23,27−30,32,34−41^ However, the TOL corrosion inhibition of imidazoline has not been reported by any research group, especially under simulated pipeline conditions. Therefore, the present work aims to develop a fatty acid imidazoline-based inhibitor using a facile solvent-free synthesis method between TOFA and DETA. An active VCI could be formulated for both BOL and TOL corrosion mitigation in oil and gas applications. The chemical identity of the as-synthesized imidazoline was verified using NMR, Fourier transform infrared spectroscopy (FTIR), and mass spectrometry. For the first time, its suitability for use as an effective VCI for carbon steel was assessed in simulated TOL test conditions of oil and gas pipelines (1 wt % NaCl with addition of 500 ppm acetic acid under a CO_2_ atmosphere). The inhibition efficacy was examined using both weight loss and electrochemical (electrochemical impedance spectroscopy (EIS), potentiodynamic polarization (PDP), and linear polarization resistance (LPR)) measurements. Adsorption isotherm studies and surface screening tests were also performed using scanning electron microscopy (SEM), energy-dispersive X-ray analysis (EDAX), and contact angle analysis.

## Experimental Section

### Materials

SYLFAT FA1 tall oil fatty acid (S-TOFA), a long carbon chain (C18) of relatively high unsaturation with the acid functionality of a carboxyl group (−COOH), was obtained from Brighten Polytrading Co., Ltd. DETA (99%), sodium chloride (99.5%), and anhydrous ethyl alcohol (99.9%) were purchased from Sigma-Aldrich, Honeywell Fluka, and Daejung, respectively.

### Synthesis of 2-(8-Heptadecenyl)-2-imidazoline-1-ethanamin (S-Imd)

**Figure 1:**
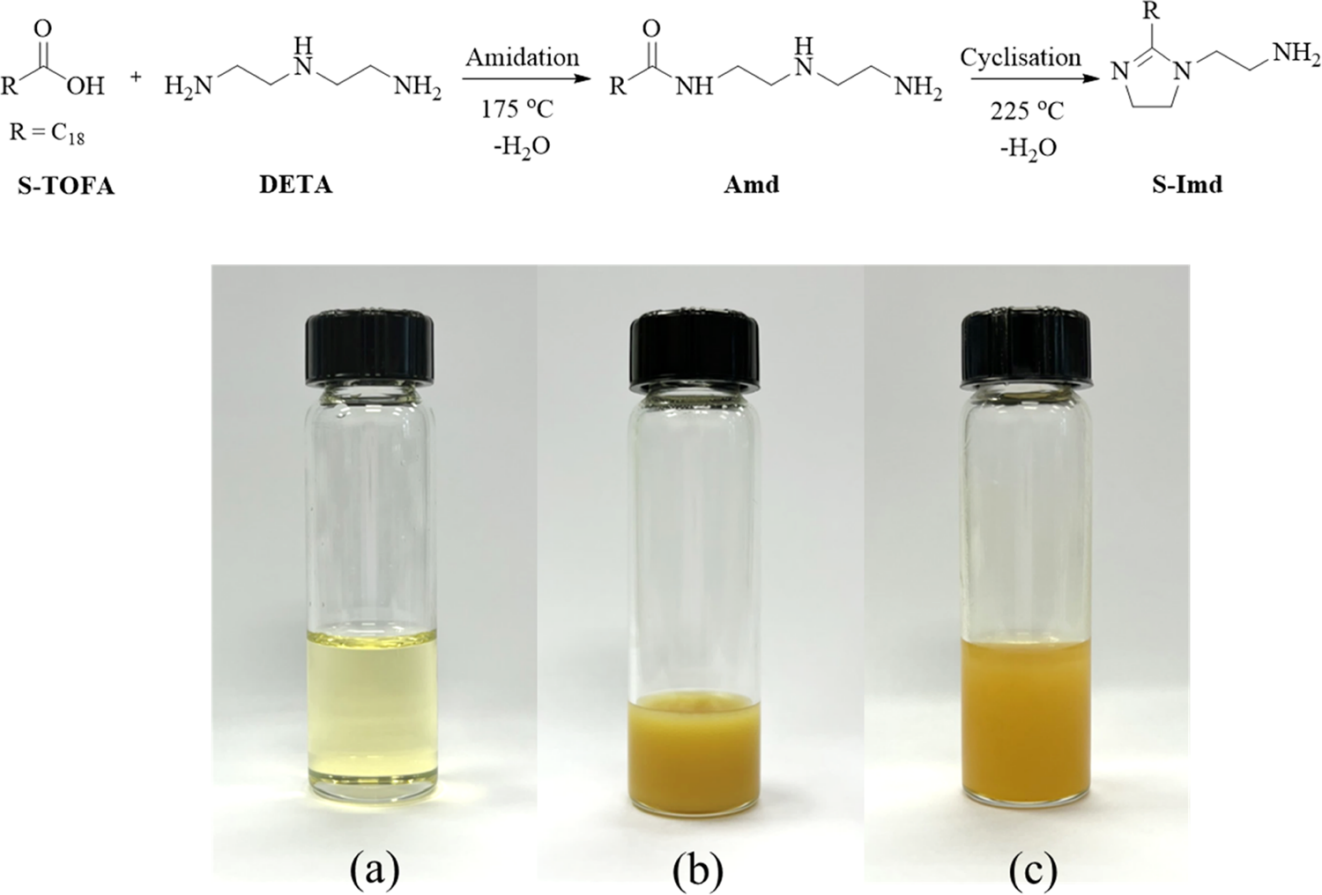
Reaction scheme of TOFA/DETA imidazoline synthesis and the physical appearance of (a) S-TOFA, (b) S-Imd, and (c) S-Imd in a 25 wt % ethanol solution.

Synthesis of fatty acid imidazolines involved a two-stage reaction including (i) amidation and then (ii) cyclization to form an imidazoline ring, as illustrated in Figure 1. The synthesis process was adapted from a previous publication.^26^ First, the S-TOFA (10 mmol) was added to a 3-necked round-bottomed flask with an installation of a Dean–Stark trap. The S-TOFA was heated to 60 °C. The DETA (12 mmol) was added dropwise to the mixture. The mixture was stirred for 3 h with continuous reflux at 175 °C. After the first-step amidation, the mixture’s color slightly changed to dark yellow. The temperature was increased to 225 °C under reduced pressure (0.01 mbar) for cyclization, which lasted for 1 h. Finally, the product (S-Imd) was obtained as a yellow wax in 85% yield. FTIR (neat): ν_max_ 3289, 3007, 2922, 2853, 1647, 1547, 1457, 1264, 986, 734 cm^–1^; ^1^H NMR (500 MHz, CDCl_3_) δ: 5.34–5.24 (m, 2H), 3.62–3.60 (m, 2H), 3.32–3.28 (m, 2H), 3.20 (br s, 2H), 2.72–2.69 (m, 4H), 2.12 (t, *J* = 7.5 Hz, 2H), 2.01–1.93 (m, 4H), 1.57–1.54 (m, 2H), 1.31–1.20 (m, 20H), 0.82 (t, *J* = 4.7 Hz, 3H); ^13^C NMR (125 MHz, CDCl_3_) δ: 173.8, 130.0, 129.8, 129.5, 127.9, 127.7, 60.6, 51.0, 48.6, 38.9, 36.5, 31.7, 31.3, 29.6, 29.53, 29.48, 29.4, 29.2, 29.0, 27.0, 25.6, 25.52, 25.46, 25.3, 22.5, 22.4, 13.94, 13.90; HRMS (ESI) *m*/*z*: (M)^+^ Calcd for C_22_H_43_N_3_ 349.3457; found 349.3203. λ_max_ = 258 nm; and melting point = 50.3 °C. Solubility in organic solvents (30 mg/mL at 25 °C): EtOH, acetone, CHCl_3_, and toluene. Figure 1b shows the resulting imidazoline product compared with pristine S-TOFA (Figure 1a). At room temperature, S-Imd became a yellow solid or wax with poor solubility in water and low volatility. In the evaluation of corrosion efficacy, S-Imd was mixed with 25 wt % ethanol to improve its suitability for use as an effective VCI in oil and gas applications.

### Characterization of S-Imd

Characterization of the imidazoline molecular structure was achieved with a ^1^H NMR spectrometer (500 MHz, Bruker). The functional groups of S-TOFA, DETA, and S-Imd were assessed using an attenuated total reflectance-FTIR spectrometer (Nicolet iS50) with wavenumbers ranging from 4000 to 600 cm^–1^. The mass of S-Imd was evaluated by a mass spectrometer (Bruker Q-TOF). Absorbance of S-Imd was determined by a UV–visible spectrometer (Spectroquant Pharo 300). The melting point of S-Imd was assessed using a thermal analyzer (METTLER/DSC1).

In the evaluation of corrosion efficacy, electrochemical measurements were carried out using a potentiostat/galvanostat (Metrohm Autolab Model PGSTAT2) with a electrocatalysis rotating ring-disk electrode (RRDE) cell, controlled and analyzed by Nova software. The electrochemical tests were performed using a standard three-electrode cell system consisting of platinum as a counter electrode, a carbon steel specimen (X65 rotating disc) as a working electrode (WE), and silver/silver chloride as a reference electrode. Electrochemical experiments were performed in a 1 wt % NaCl solution with addition of 500 ppm acetic acid under a CO_2_ atmosphere with a flow rate of 20 mL/min at room temperature. The dosage of imidazoline was varied between 50 and 500 ppm.^42^ For all electrochemical tests, the WE was immersed in the test solution for 1 h to establish at least a pseudo-steady-state open-circuit potential (OCP).

For EIS studies, the metal coupon was immersed in 1 wt % NaCl solution in the absence and presence of the inhibitor, which allowed establishment of a steady-state OCP. EIS was obtained by superimposing a sinusoidal alternating current (AC) signal with an amplitude of 10 mV and over the frequency range from 100,000 to 0.01 Hz. EIS measurements were carried out at a controlled immersion time of 5 min for all samples. Nova software was used for the analysis of impedance curves.

For PDP measurements, the voltage was scanned from a cathodic potential of −250 mV to an anodic potential of +250 mV, with a scanning rate of 0.2 mV/s. Extrapolation was used to determine polarization parameters such as corrosion potential (*E*_corr_), current density (*i*_corr_), anodic and cathodic Tafel slopes (β_a_ and β_c_), and polarization resistance (*R*_p_) from the obtained polarization curves. LPR was measured by polarizing the X65 rotating steel electrode from −10 to +10 mV versus OCP with a scan rate of 0.125 mV/s. *R*_p_ was obtained from the slope of the potential–current graph in the vicinity of *E*_corr_.

**Figure 2:**
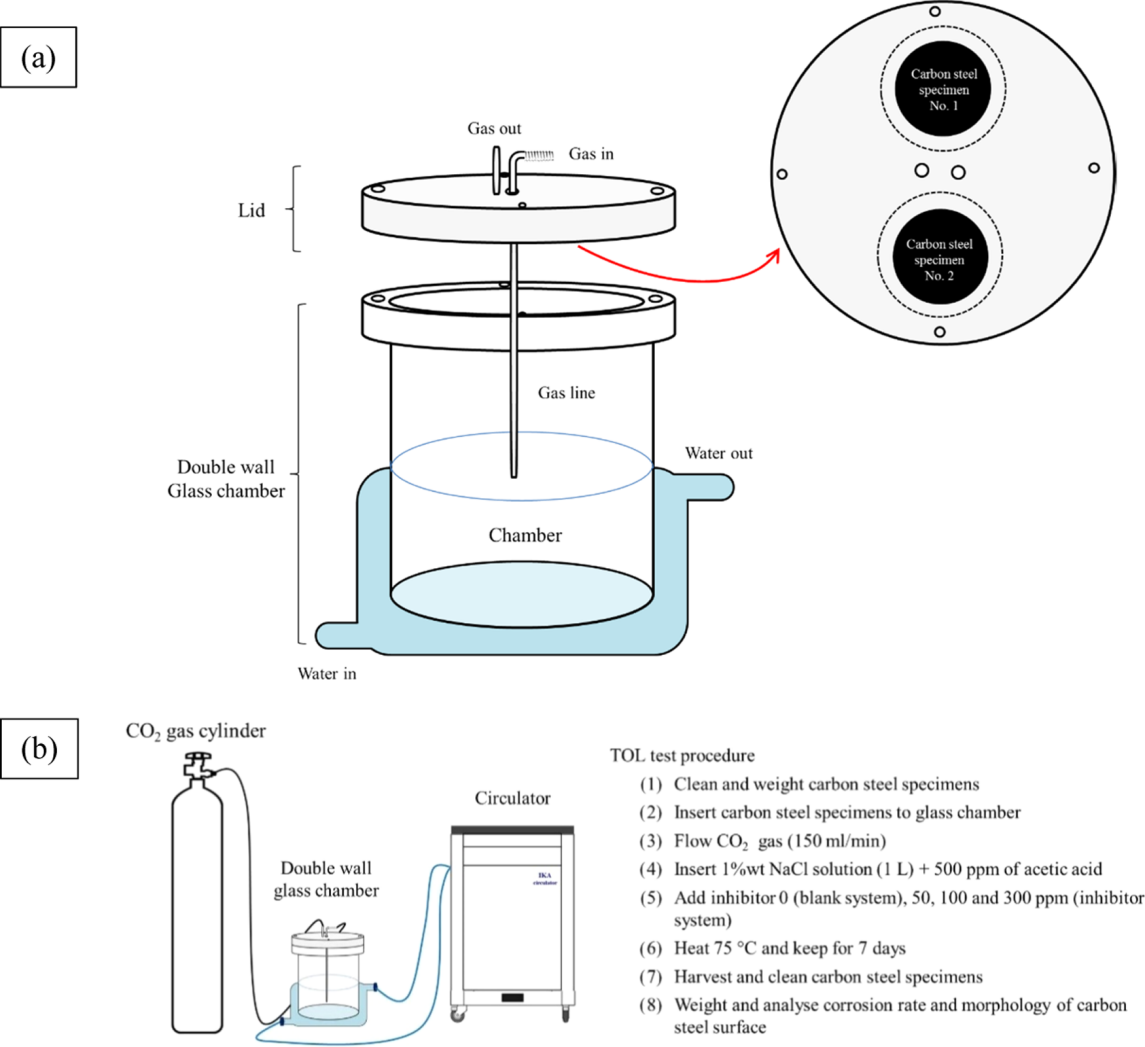
(a) TOL glass reactor design and (b) TOL test procedure.

In the TOL corrosion tests, carbon steel sheets (API 5L grade-X65, Ø 40 mm) were degreased by rinsing them with ethanol (99.9%), polishing with SiC paper (1000 mesh), sonicating with 75% v/v ethanol for 3 min, and finally drying using an air gun. The initial weight of the test specimens was recorded before use. TOL corrosion was measured via a weight loss technique for 7 days in a simulated pipeline environment in a glass reactor, as shown in Figure 2a. The carbon steel specimens were examined in 1 wt % NaCl solution with addition of 500 ppm acetic acid in the absence and presence of the inhibitor at 75 °C controlled by a circulator. The inhibitor was prepared by dissolving the S-Imd in ethanol to enhance vapor pressure and increase its solubility in a water phase. The suitable level of S-Imd was studied including 0 (blank system), 50, 100, and 300 ppm. Additionally, the amount of oxygen that was present and entered the test reactor and the test solution was minimized by continuously flowing CO_2_ gas into the reactor during the tests with a fixed flow rate of 150 mL/min. The TOL test procedure is illustrated in Figure 2b.

After the test, the specimens were removed from the TOL glass reactor, cleaned with distilled water, and then immersed in an aqueous pickling solution (ASTM G1 Standard Practice)^43^ to remove the corrosion products deposited on the specimen surfaces that occurred during the corrosion test. The specimens were subsequently washed with distilled water, dried, and weighed to determine the final weight. The corrosion rate (CR) is calculated based on weight loss measurements, as expressed in eq 1.^44^
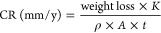
1where weight loss is in grams, *K* is the conversion factor of 8.76 × 10^4^ (in mm/y), ρ is the density of the sample in g/cm^3^ (density of steel = 7.87 g/cm^3^), *A* is the area of the specimen (cm^2^), and *t* is the time of exposure (h).

The surface morphology of the carbon steel specimens after TOL testing was investigated by using SEM (HITACHI/SU5000) coupled with EDAX. The elemental composition of the corrosion products or the adsorbed imidazoline films on the specimen surfaces was also examined. The contact angles of water droplets on those specimens were measured at 25 °C using a contact angle goniometer (DataPhysics, OCA40) to investigate their surface hydrophobicity/hydrophobicity and roughness.

## Results and Discussion

### Structure of S-Imd

**Figure 3:**
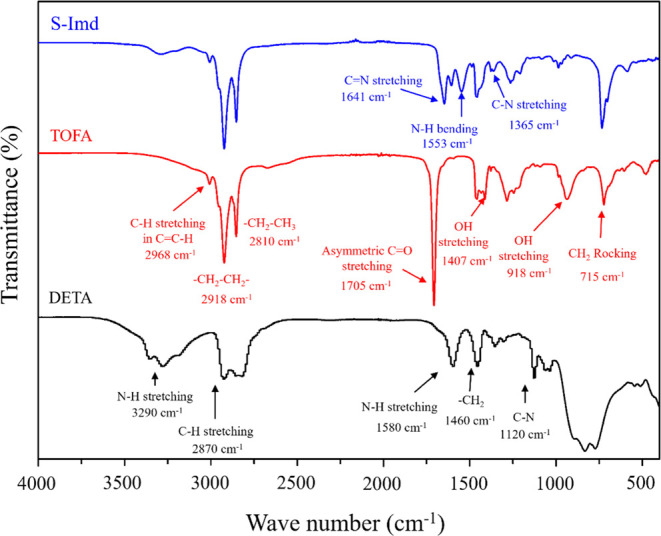
FTIR spectra of DETA, S-TOFA, and S-Imd.

The FTIR spectra of DETA, S-TOFA, and S-Imd are shown in Figure 3. For DETA, characteristic peaks are located at around 3290, 2870, 1580, 1460, and 1120 cm^–1^ corresponding to N–H stretching, C–H stretching, N–H stretching, −CH_2_, and C–N, respectively. The absorption bands of the S-TOFA compound can be found at 2968, 2918, 2810, 1705, and 715 cm^–1^ along with 1407 and 918 cm^–1^, which are ascribed to C–H stretching, −CH_2_–CH_2_–, −CH_2_–CH_3_, C=O, CH_2_ rocking, and −OH stretching. For the S-Imd sample, the absorption bands of the N–H group in DETA (at 3290 cm^–1^) and of the carbonyl ester (C=O) group in S-TOFA (at 1705 cm^–1^) vanished, indicating the formation of imidazoline molecules. The imidazoline spectrum also presented new absorption bands at 1641, 1553, and 1365 cm^–1^, which were ascribed to C=N, N–H, and C–N bonds, respectively. This agreed well with characteristic absorption peaks for imidazoline reported in the literature.^21,45−47^

**Figure 4:**
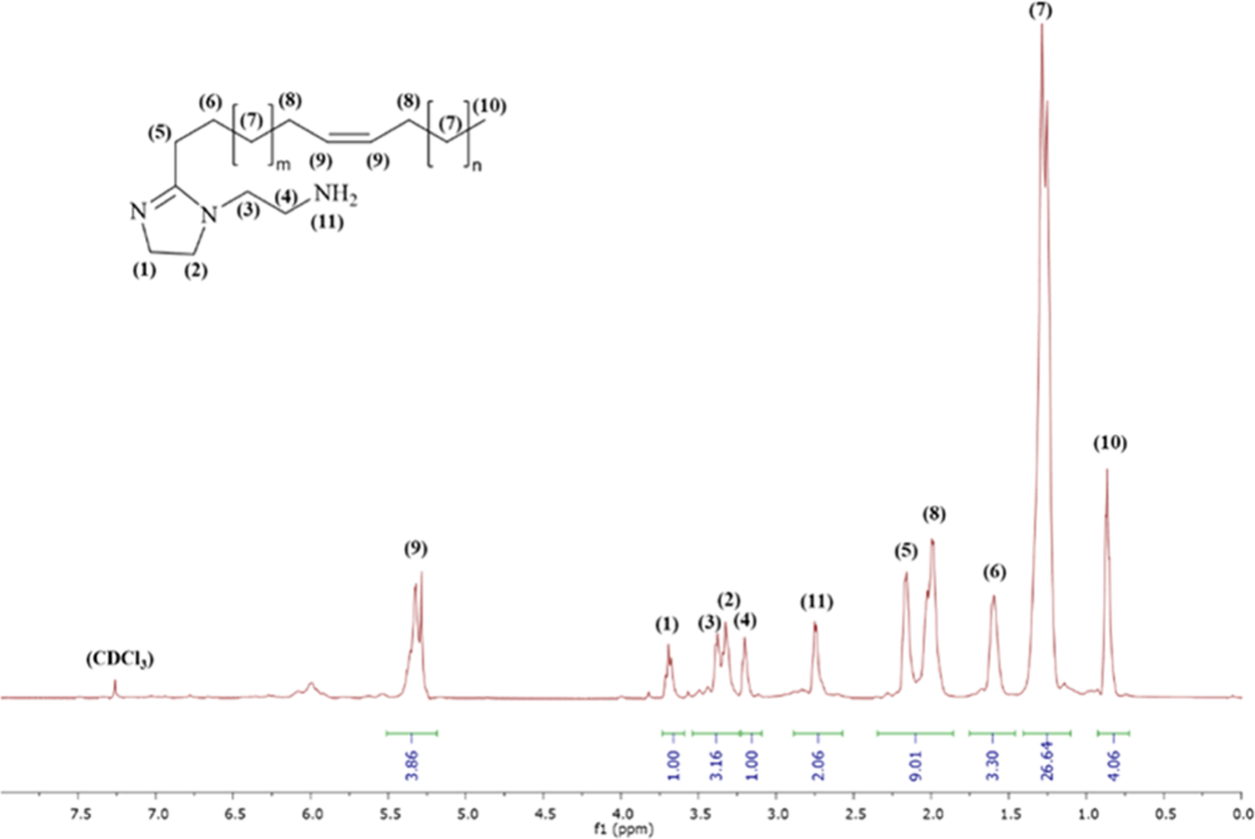
^1^H NMR spectra of S-Imd.

Structural characterization of S-Imd was carried out using an NMR technique, and the results are shown in Figure 4. The ^1^H NMR results confirmed successful synthesis of imidazoline with the presence of an equivalent methylene group in the imidazoline ring at a δ-value of 3.30 and 3.70 ppm.^24,47^ The peak δ-value of 5.31 ppm corresponded to an unsaturated bond in the hydrocarbon chain (m, 2H, −CH=CH−).^18^ The proton signal originating from the pendant group of the imidazoline molecule (i.e., CH_2_ attached to the carbon of imidazoline) shifted to 3.41, 3.18, and 2.20 ppm.^24^ Additionally, the peaks at δ-values of 0.86, 1.36, 1.60, and 1.98 ppm represented protons in the hydrocarbon chain.^18^ (^13^C NMR spectra, mass spectra, and UV–visible spectra in Supporting Information Figures S1–S3.)

**Figure 5:**
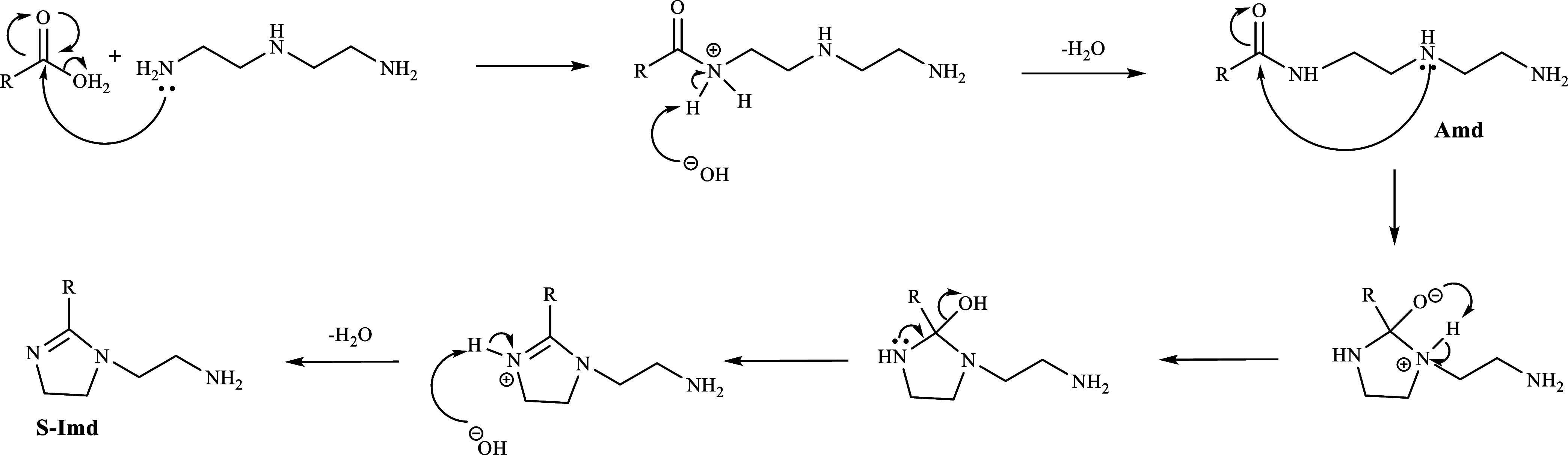
Mechanism of S-TOFA/DETA imidazoline synthesis.

A mechanism of the synthesis reaction for the fatty acid imidazoline is demonstrated in Figure 5. First, the carbonyl group of tall oil fatty acids was attacked by a lone pair electron of nitrogen atoms in the amine group. After that, a water molecule was eliminated, resulting in the formation of *N*-(2-((2-aminoethyl)amino)ethyl)octadec-8-enamide (Amd). Next, the S-Imd was obtained from the intramolecular cyclization reaction of the Amd intermediate, which was initiated by a lone pair electron of N atoms of secondary amine attacking the carbonyl group of Amd. Finally, a water molecule was eliminated, yielding S-Imd.

### Corrosion Inhibition Studies

#### OCP Studies

**Figure 6:**
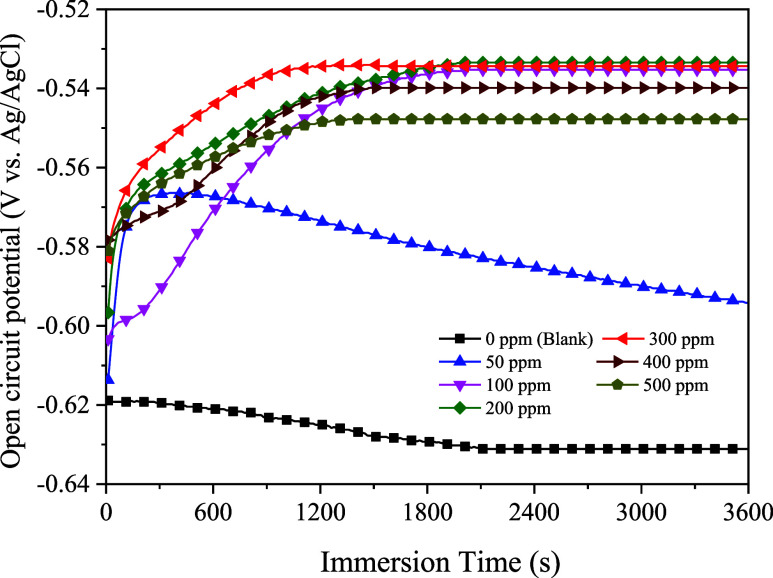
Variation of the OCP over immersion time of the carbon steel WE in 1 wt % NaCl containing different concentrations of S-Imd at 25 °C.

The working electrodes were allowed to corrode in a blank solution with and without the presence of different concentrations of the as-synthesized imidazolines at the OCP. Figure 6 shows the *E*_ocp_ values as a function of immersion time. In the blank system, the *E*_ocp_ values moved toward a more negative state and potentially had the lowest *E*_ocp_ value of −0.6311 V vs Ag/AgCl due to the breakdown of the oxide film on the carbon steel surface.^24,48,49^ The negative values of *E*_ocp_ curves indicated the corrosive nature of the blank solution due to the presence of negative chloride ions on the WE in the test medium.^50^

In solutions with inhibitors added, the *E*_ocp_ values at various inhibitor doses moved toward more positive values because the inhibitor molecules could form as a protective film layer on the WE. It was found that the protective film layer was probably unstable at a very low dose of 50 ppm (0.14 mM) as the *E*_ocp_ decreased gradually with an increased immersion time. At higher concentrations, the *E*_ocp_ values increased rapidly to their plateau as a sign of stability due to continuous film formation at approximately −0.5353, −0.5335, −0.5344, −0.5399, and −0.5478 V (vs Ag/AgCl) for imidazoline doses of 100 ppm (0.29 mM), 200 ppm (0.57 mM), 300 ppm (0.85 mM), 400 ppm (1.14 mM), and 500 ppm (1.43 mM), respectively. Similar results were reported in the literature.^24,49,51^ It was suggested that the *E*_ocp_ observed in the inhibited system should move to more positive values and an immersion time of 1 h was required to establish stable formation of an inhibitor protective layer during the electrochemical measurements.^51,52^

Among various concentrations of S-Imd inhibitor, 300 ppm was found to be the optimal concentration for corrosion inhibition of carbon steel surfaces immersed in a 1 wt % NaCl solution as the *E*_ocp_ of the inhibited system reached its plateau most quickly.^53^ An increased inhibitor concentration resulted in the formation of more micelles in the bulk solution rather than the adsorption of molecules of the inhibitor on metal surfaces (Figure 6).

#### EIS Studies

**Figure 7:**
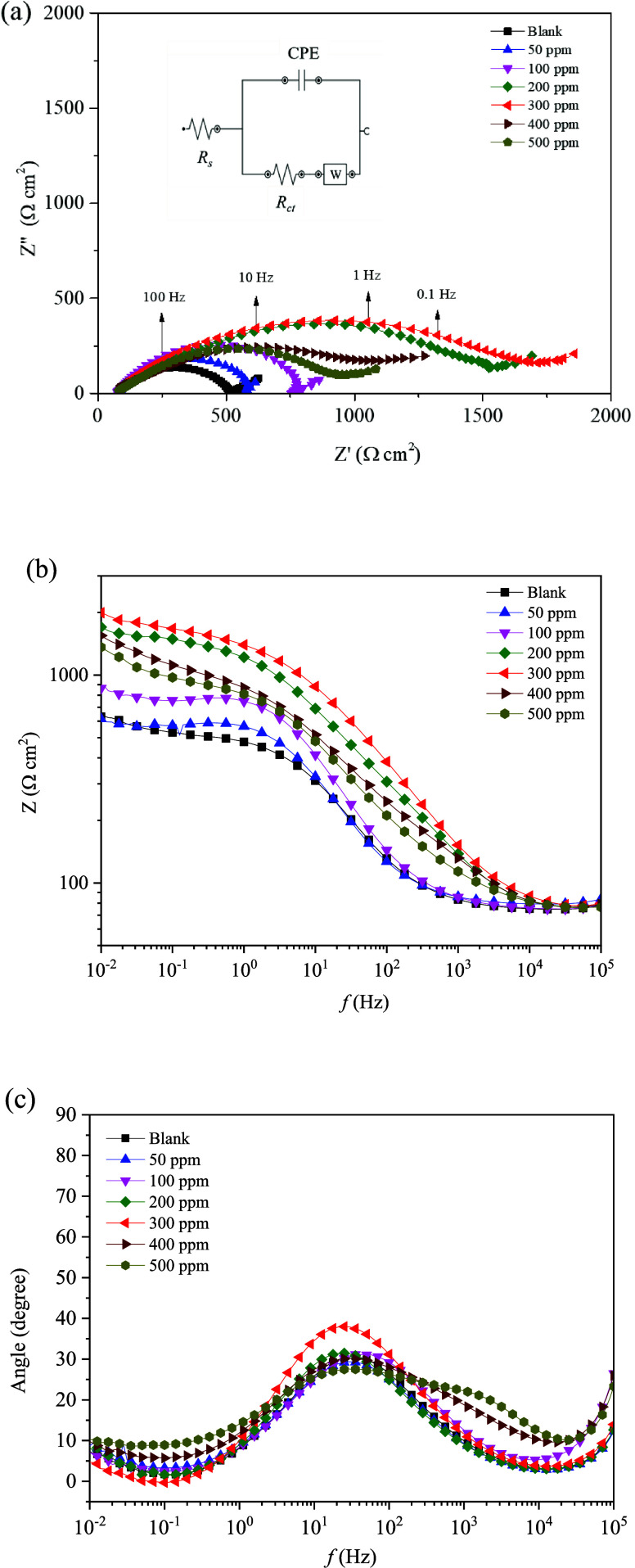
Electrochemical impedance spectra of carbon steel in a 1 wt % NaCl solution containing various concentrations of S-Imd: (a) Nyquist plot and equivalent circuit model used to fit the EIS experimental data. (b) Bode modulus and (c) phase angle plots at 25 °C.

The EIS technique could provide information about the kinetics of electrode processes and the surface properties of the carbon steel studied. Formation and destruction of inhibitor films and the inhibition efficacy of S-Imd were evaluated by EIS on a carbon steel WE in a 1 wt % NaCl solution. Figure 7a shows representative Nyquist plots for the impedance response of carbon steel samples in the absence or presence of S-Imd at different doses. As can be seen from the Nyquist plots, the shape of all impedance diagrams was the same for all studied conditions, both uninhibited and inhibited in 1 wt % NaCl solutions. This implied that the corrosion mechanism on the metal surface was unchanged even in the presence of inhibitors.^51^ Each plot consisted of one depressed semicircle, indicating nonideal electrochemical behavior at the interface between the heterogeneities of the electrode surface and the electrolyte solution. In addition to a depressed semicircle, a Warburg impedance component could be observed at low frequencies, attributed to a diffusion limitation of metal ions or the ingress of the electrolyte through the corrosion product/inhibitor layer. The arc diameters of the inhibited systems with respect to the real part (*X*-axis) were larger than those of the blank system and were increasingly higher with greater inhibitor concentrations. This was caused by the inhibitive action of a protective layer that mitigated the corrosion processes. The charge transfer process was retarded by adsorption of an inhibitor film on the surface of carbon steel that replaced water molecules.^6,18,35,45^ Charge transfer resistance was found to be higher with increasing inhibitor concentrations from 50 to 300 ppm. However, a saturation point was reached at higher concentrations of 400 and 500 ppm, as can be seen from the decreased arc diameters. When the inhibitor concentration is excessive, the preadsorbed inhibitor species begins to interact with the nonadsorbed species, resulting in desorption of the inhibitor film. This study confirmed that the optimal dose of S-Imd was relatively low, 300 ppm, compared to VCI doses of up to 5000 ppm that are typically applied in oil and gas applications.^53,54^

The corresponding Bode modulus diagrams and Bode phase angle diagrams are presented in Figure 7b,c. The Bode modulus of the impedance data reveals a single time constant for all the samples (i.e., one peak in the phase shift curves), which is consistent with the Nyquist plots. The impedance (*Z*) was shifted to higher values with an increasing concentration of S-Imd to 300 ppm, which was more significant in the low-frequency region. This indicated that S-Imd was effective in preventing the dissolution of carbon steel in a corrosive solution. The highest inhibition was noted for 300 ppm imidazoline, which is comparatively lower than previous reports.^2,41,50^ Phase angle and frequency plots in Figure 7c reveal a single peak for all conditions. The phase angle increased with the addition of imidazoline, thus confirming the effective adsorption of this inhibitor onto the carbon steel surfaces. As in the Nyquist plots, desorption of the preadsorbed inhibitor film at high concentrations, 400 and 500 ppm, led to decreased phase angles and *Z* values. The shift of the Bode modulus and an increase in the Bode phase angle of the EIS curve confirmed the coverage of absorbed imidazoline molecules on carbon steel surfaces.

An equivalent circuit is used to model the electrochemical behavior and calculate the parameters of interest. Interpretation of the electrochemical behavior from the EIS spectra required an appropriate physical model that accurately represents the electrochemical process and system. An equivalent circuit model was proposed for this purpose that considers a carbon steel/solution reaction such as double-layer capacitance, solution resistance, charge transfer resistance, and Warburg effect. The impedance results were obtained by fitting the equivalent circuit model (see the inset of Figure 7a). The equivalent circuit diagram perfectly fitted the impedance data recorded under uninhibited and corrosion-inhibited conditions. The EIS experimental data of the uninhibited (blank) and the inhibited systems were fitted with a single time constant model consisting of a solution resistance (*R*_s_), a charge transfer resistance (*R*_ct_), and a constant phase element (CPE). The capacitive arcs in Figure 7a were not ideal semicircles (nonideal capacitance behavior persists at the solid and liquid interface). Thus, the CPE represents the capacitance because it is influenced by not only pure capacitance but also surface characteristics such as surface roughness, discontinuity of the inhibitor layer adsorbed on the metal surface, and inhomogeneity in the conductance or dielectric constant. Moreover, the Warburg impedance (*W*) was related to the diffusion of an electroactive species through an electrode surface. This was fitted in the Nyquist plot in the low-frequency band as a straight line with 45° angle.^55,56^

Impedance of the CPE was calculated using eq 2.^2,19,35^
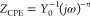
2where *Y*_0_ is the CPE constant or a proportionality coefficient, ω is the angular frequency (in rad/s),  is the imaginary number, and *n* is a CPE exponent that is a measure of surface heterogeneity (−1 ≤ *n* ≤ 1). CPE represents pure resistance (*R*) for *n* = 0, a pure capacitance (*C*) for *n* = +1, a pure inductance (*L*) for *n* = −1, or a Warburg impedance for *n* = 0.5.^2,19^ This work used a *Y*_0_ value that was converted into double-layer capacitance *C*_dl_ using eq 3.^40^
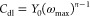
3where ω_max_ = 2π*f*_max_ and *f*_max_ is the frequency at which the imaginary component of the impedance is maximum. The inhibition efficiency, % η_EIS_, is given by eq 4.^19,52^
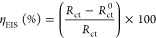
4where *R*_ct_^0^ and *R*_ct_ are the charge transfer resistances in the blank and inhibited systems, respectively.

Table 1 lists fitted EIS parameters such as *R*_s_, *Y*_0_, *R*_ct_, *C*_dl_, and % η_EIS_ obtained in the absence and presence of the S-Imd inhibitor at different dosages. Similar to what is obviously seen by the EIS spectra, the data clearly reveal that the increasing of S-Imd concentration from 0 to 300 ppm reduces the *C*_dl_ values but enhances the *R*_ct_ values as well as the resulting % η_EIS_. There was a large variation in the *R*_ct_ values between the inhibited and uninhibited systems, whereas the *R*_s_ values were relatively unchanged. The increase in the *R*_ct_ values confirmed that there was a protective film on the carbon steel surface, which acted as a barrier to mass and charge transfer. This implied that the S-Imd molecules adsorbed on the surface of the carbon steel samples. The highest *R*_ct_ value was 336.5 Ω·cm^2^ at an imidazoline concentration of 300 ppm, corresponding to the greatest corrosion inhibition performance, 70.5%, after 5 min of exposure. This means that the S-Imd molecules can quickly form a protective layer on metal surfaces at this optimal concentration, which become effective much faster than reported in previous work. For instance, only 20–52% inhibition efficiency was obtained with thiosemicarbazone derivatives after a longer exposure time, 2 h.^57^ Furthermore, the decrease in the *C*_dl_ values with increasing inhibitor concentration is attributed to the formation of the dielectric constant by the adsorptive layer. It can be otherwise explained by the depletion of double-layer capacitance at the metal surface/solution interface because organic molecules get adsorbed onto the metal surface by replacing the preadsorbed water molecules.

**Table 1:** Fitted EIS Parameters for Carbon Steel in 1 wt % NaCl Containing Various Imidazoline Concentrations at 25 °C^a^,^b^

concentration (ppm)	*R*_s_ (Ω·cm^2^)	*Y*_0_ (mΩ·s*^n^*/cm^2^)	*Y*_0_(*W*) (mΩ·s^0.5^/cm^2^)	*R*_ct_ (Ω·cm^2^)	*C*_dl_ (μF/cm^2^)	Θ (deg)	χ*^2^*	η_EIS_ (%)
0	14.7 ± 0.1	77.9 ± 1.9	273.9 ± 12.5	99.3 ± 0.5	113	30.7	0.086	
50	16.5 ± 0.1	26.0 ± 0.4	258.5 ± 3.4	114.1 ± 0.2	51.9	29.4	0.090	13.0
100	14.8 ± 0.1	20.1 ± 0.4	234.9 ± 8.40	148.9 ± 0.2	32.9	31.2	0.091	33.3
200	15.8 ± 0.1	16.6 ± 0.6	212.1 ± 7.2	299.1 ± 0.7	9.4	31.4	0.037	66.8
300	16.4 ± 0.1	15.1 ± 0.4	82.2 ± 0.9	336.5 ± 0.5	5.8	38.0	0.007	70.5
400	15.8 ± 0.2	16.8 ± 0.3	66.6 ± 0.3	213.7 ± 0.4	11.1	30.2	0.031	53.5
500	15.8 ± 0.1	15.4 ± 0.6	83.2 ± 1.5	190.3 ± 2.3	12.9	27.6	0.013	47.8

a Θ (deg) = phase shift.

b χ^*2*^ = goodness of fit.

#### PDP and LPR Studies

**Figure 8:**
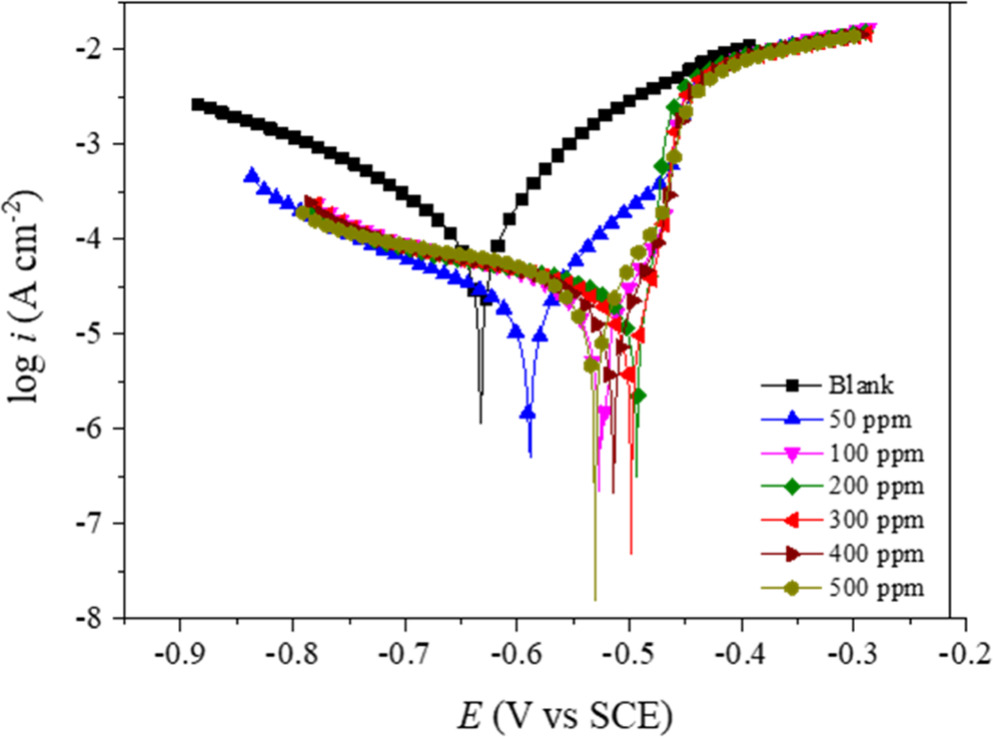
PDP plots for carbon steel in a 1 wt % NaCl solution containing various concentrations of the as-synthesized imidazoline at 25 °C.

A plot of applied potential (*E*) vs current density (log *i*) or a PDP curve for carbon steel in 1 wt % NaCl containing different concentrations of S-Imd is shown in Figure 8. In the absence and presence of S-Imd, the cathodic branches exhibit Tafel lines, implying that the addition of the inhibitor does not alter the mechanism of H^+^ reduction. The H^+^ reduction occurs primarily through the electron transfer process.^58^ As for the anodic curves, two main Tafel slopes can be noticed. First, the anodic current densities increase as the potential becomes more anodic. After the potential reaches the desorption potential *E*_dsp_, one may notice a sharp rise of anodic current densities followed by flatness. This behavior may be related to the displacement of adsorption–desorption equilibrium toward the desorption of the S-Imd molecule from the carbon steel substrate.^59^ The *E*_corr_ values in inhibited systems were shifted to a more anodic condition (less negative value) with respect to that of the blank solution. This indicates that S-Imd has a greater inhibitive influence on the anodic corrosion half-reactions than on the cathodic corrosion half-reactions. The difference between *E*_corr_ of blank and inhibited systems is greater than 85 mV, implying that S-Imd functioned as a mixed-type inhibitor predominantly of the anodic reaction.^50,52^ The corrosion inhibition effect of S-Imd was reflected in the displacement of the anodic and cathodic curves to lower current densities or remarkably shifting them to a more negative potential in comparison to the blank sample, verifying its inhibition efficacy in rendering anodic and cathodic reactions. Nevertheless, the displacement was rather more pronounced for anodic than for cathodic reactions. In light of this result, S-Imd acted as a mixed-type inhibitor with a predominantly anodic control, similar to reports of previous studies.^3,6,18,21,24,40,60^

**Figure 9:**
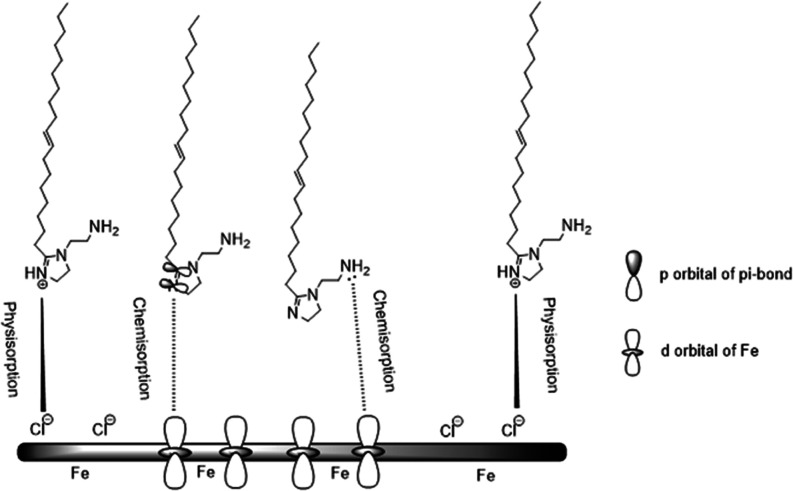
Adsorption mechanism of S-Imd with a carbon steel surface.

The absorption processes of S-Imd on the carbon steel surface are based on its chemical structure containing π-electrons in the ring, unshared electrons in the heteroatom, and a double bond present in C=N. The absorption mechanisms of S-Imd on a carbon steel surface could be considered physisorption or chemisorption and a mixed type of both physisorption and chemisorption (Figure 9). First, physisorption is the interaction of two charged species. Later, chemisorption occurs through a charge-transferring process, forming a very stable film. The adsorption layer of S-Imd on the metal surface is a single layer structure according to Langmuir’s isotherm and thermodynamic theory. The long hydrocarbon chain in the S-Imd structure provides a barrier to water and chloride ingress. It can be seen from Figure 9 that all of the parts in S-Imd play a great role in inhibiting the corrosion processes (both anodic and cathodic reactions) by changing the surface status of carbon steel samples in the corroding solution.

Additionally, a PDP curve can be used to determine the values of various parameters such as *E*_corr_, β_a_, β_c_, *i*_corr_, and PDP inhibition efficiency (% η_PDP_), as listed in Table 2. The % η_PDP_ values can be obtained using eq 5:^4,19,35^
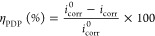
5where *i*_corr_^0^ and *i*_corr_ represent the values of corrosion current densities for carbon steel in the absence and presence of the inhibitor, respectively.

**Table 2:** Corrosion Parameters for Carbon Steel in 1 wt % NaCl Solutions in the Absence and Presence of Different Concentrations of S-Imd at 25 °C from PDP and LPR Methods

	PDP								LPR		
concentration (ppm)	OCP (V vs Ag/AgCl)	*E*_corr_ (mV vs Ag/AgCl)	*i*_corr_ (μA/cm^2^)	β_a_ (mV/dec)	–β_c_ (mV/dec)	*R*_p_ (Ω·cm^2^)	CR (mm/y)	η_PDP_ (%)	*R*_p_ (Ω·cm^2^)	CR (mm/y)	η_LPR_ (%)
0	–0.665 ± 0.005	664.7 ± 5.4	45.42 ± 0.15	40.62 ± 0.36	40.74 ± 0.25	267.87 ± 9.73	0.370		149.34 ± 4.71	0.439	
50	–0.593 ± 0.005	592.6 ± 5.4	5.99 ± 0.26	31.44 ± 0.29	47.29 ± 0.24	1390.54 ± 2.16	0.069	86.8	386.52 ± 9.01	0.170	61.4
100	–0.515 ± 0.010	515.3 ± 10.4	6.00 ± 0.32	32.50 ± 1.07	46.35 ± 0.40	1451.53 ± 54.26	0.068	86.8	424.36 ± 9.30	0.155	64.8
200	–0.489 ± 0.014	488.6 ± 13.8	4.36 ± 0.18	13.12 ± 0.26	17.26 ± 0.21	700.86 ± 70.89	0.049	90.4	1256.84 ± 10.73	0.052	88.1
300	–0.494 ± 0.007	493.8 ± 7.00	3.83 ± 0.08	17.47 ± 0.34	25.39 ± 0.28	1195.29 ± 8.36	0.044	91.6	1266.47 ± 8.40	0.052	88.2
400	–0.521 ± 0.020	521.0 ± 20.4	4.44 ± 0.07	22.19 ± 0.24	34.22 ± 0.32	1317.97 ± 26.78	0.052	90.2	1128.95 ± 23.73	0.058	86.8
500	–0.527 ± 0.004	527.2 ± 4.3	4.69 ± 0.19	23.42 ± 0.33	26.81 ± 0.57	1194.71 ± 24.15	0.054	89.7	1201.56 ± 19.23	0.055	87.6

The *i*_corr_^0^ value was 45.42 ± 0.15 μA/cm^2^ in the blank solution, whereas the inhibited system containing 50, 100, 200, 300, 400, and 500 ppm imidazoline had *i*_corr_ values of 5.99 ± 0.26, 6.00 ± 0.32, 4.36 ± 0.18, 3.83 ± 0.08, 4.44 ± 0.07, and 4.69 ± 0.19 μA/cm^2^, respectively. It is very clear that the presence of S-Imd in a 1 wt % NaCl solution significantly reduced corrosion current density, leading to a reduced corrosion rate for carbon steel. Additionally, the inhibition efficiency of S-Imd increased to its maximum value of 91.6% as its concentration was increased to 300 ppm. At higher concentrations (over 300 ppm), the preadsorbed species were desorbed and thus adversely affected corrosion inhibition efficacy.

Corrosion inhibition of carbon steel in a 1 wt % NaCl solution by S-Imd was also evaluated using an LPR approach. The LPR technique provides a rapid corrosion analysis in which the WE is polarized by a very small potential perturbation of ±10 mV versus OCP. The *R*_p_ is defined by the slope of the linear portion of a voltage-versus-current curve near the *E*_corr_ in Figure 8. This *R*_p_ value was then used to calculate inhibition efficiency as given in eq 6.
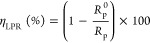
6*R*_p_^0^ and *R*_p_ are the polarization resistance values in the absence and presence of the inhibitor, respectively. The trend of the *R*_p_ values as a function of the inhibition efficacy was the same as that for the PDP results. As can be seen in Table 2, the *R*_p_ value increased in the presence of S-Imd. With increased imidazoline concentration, its excellent efficacy for corrosion mitigation of carbon steel in a 1 wt % NaCl solution was verified. A maximum *R*_p_ value, 1266.47 Ω·cm^2^, was obtained at the optimum imidazoline concentration, 300 ppm. This corresponds to an inhibition efficiency of 88.2%, which is in good agreement with the results attained from the PDP analysis.

Although both EIS and PDP methods can be employed to assess the corrosion rate, polarization resistance, and inhibition efficiency of the inhibited system compared to the uninhibited one, they are different in terms of the approach in measurement and the information they provide. They are sensitive to different types of corrosion: the EIS method is more sensitive to localized corrosion, e.g., pitting and crevice corrosion, whereas the PDP method is more sensitive to uniform corrosion. Hence, there are differences in the polarization resistance and inhibition efficiency obtained by EIS and PDP methods (see Tables 1 and 2). For instance, the results obtained by the EIS method are raised by the changes in the electrical properties of a metallic material due to the localized corrosion occurring in specific locations rather than across the entire exposed surface. The interpretation of the electrochemical behavior from the EIS spectra relies on electrical characteristics including resistances, capacitors, or constant phase elements that are connected in parallel or in a series to form an equivalent circuit. The PDP method, on the other hand, provides information on the polarization resistance observed in DC circuits that obeys Ohm’s law directly and also on the inhibition efficiency of S-Imd caused by uniform corrosion.

#### TOL Corrosion Test Based on a Weight Loss Technique

**Figure 10:**
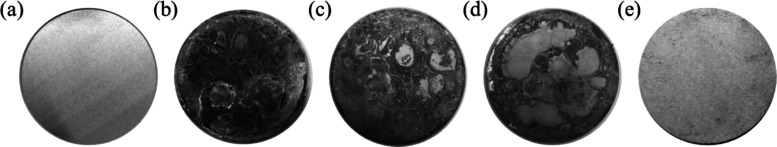
Optical images show the surface appearance of the carbon steel specimens before and after soaking in 1 wt % NaCl in the TOL corrosion test via a weight loss technique; the specimens include (a) initial, (b) blank specimen after soaked in an uninhibited solution (no inhibitor added), and (c–e) test specimens after soaking in inhibited systems containing S-Imd at different dosages of 50, 100, and 300 ppm, respectively.

Imidazoline compounds act as active volatile corrosion inhibitors with excellent corrosion–protection properties. They exhibit evaporative properties by transitioning into the gas phase and filling the entire volume with a continuous chemical cloud. When the vapor concentration reaches a saturated level, a protective film is formed on metal surfaces that mitigates corrosion processes and thus reduces corrosion rates. It can be assessed by different methods. Weight loss is a direct measurement that can be used to evaluate the TOL corrosion rate of carbon steel using S-Imd in the simulated conditions of a gas pipeline (at 75 °C under a CO_2_ atmosphere). Figure 10 shows imagery, with no color adjustment, of carbon steel surface specimens before and after use in the TOL corrosion test.

CR values and corrosion efficiency (% η_WL_) measured using a weight loss technique are given in Table 3. The CR values were reduced from 0.72 to 0.68 and 0.62 mm/y with increasing imidazoline concentrations from 0 (uninhibited system) to 50 and 100 ppm, respectively. The CR value of the inhibited system containing 300 ppm imidazoline was the lowest, 0.07 mm/y. The inhibition efficiency of S-Imd on corrosion at the carbon steel surface increased with concentration, up to 300 ppm, yielding an inhibition efficiency of up to 89.9%. This confirmed that the optimum S-Imd concentration was 300 ppm to prevent both BOL and TOL corrosion in a gas pipeline, similar to the results attained with electrochemical corrosion tests.

**Table 3:** Corrosion Results Using Weight Loss Measurements

test conditions	CR (mm/y)	η_WL_ (%)
0 ppm (blank)	0.72	
50 ppm imidazoline	0.68	4.7
100 ppm imidazoline	0.62	13.1
300 ppm imidazoline	0.07	89.9

It is noted that the corrosion efficiency of electrochemical and weight loss measurements is different because the weight loss measurement was employed to measure TOL corrosion rates of metallic specimens at which the corrosion processes were inhibited by a VCI protective film through a vaporization process. The corrosion efficiency obtained from the PDP measurement was simulated by the BOL corrosion conditions at which the metal specimen was immersed into the test solution with the presence of VCI molecules, which then formed a protective film on the metal surface.

#### Adsorption Studies

**Figure 11:**
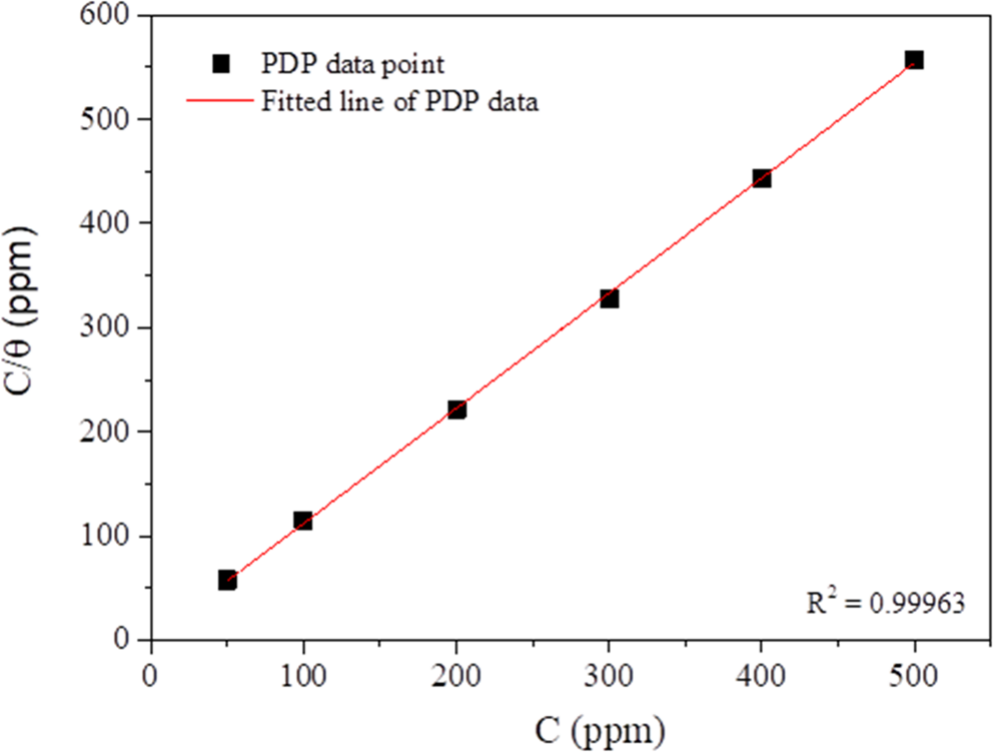
Langmuir adsorption isotherm of S-Imd for a carbon steel surface in a 1 wt % NaCl solution at 25 °C based on PDP data.

The adsorption of inhibitor molecules on the metal surface depends strongly on the following factors: (i) the surface charge of the metal surface, (ii) the charge on the inhibitor molecule, and (iii) the dipole moment of the inhibitor molecule and counterions that are specifically adsorbed on the metal surface.^61^ The surface charge on the metal surface in the corroding medium can be determined from the position of open-circuit potential with respect to the potential of zero charge (PZC).^62^ It has been reported that the surface charge of the mild steel substrate is usually positive in both the inhibited and uninhibited solutions.^63^ For example, in NaCl solution, the metal surface is positively charged at its OCP conditions. In the uninhibited system, the dissolution of metal involves successively the reversible adsorption of the Cl^–^ ions on the metal surface, releasing electrons from the adsorbed anions to the metal surface. After that, the adsorbed anions along with Fe^2+^ ions are desorbed by harvesting electrons from the Fe atoms. With the presence of inhibitor molecules in the corroding medium, the protonated inhibitor molecules at the N atoms are in equilibrium with the unprotonated form. Because of electrostatic repulsion, a positively charged molecule struggles to approach a positively charged metal surface. The positively charged inhibitor molecules can be readily adsorbed on the mild steel surface via the Cl^–^ ions. This results in the formation of a connecting bridge between the protonated inhibitor molecules and the metal surface. In addition to the physisorption process, the adsorption of the unprotonated form of inhibitor molecules can also take place through a donor–acceptor via an interaction between π electrons of double bonds or the lone pair of electrons on N atoms and the vacant “d” orbitals of surface iron atoms. In this work, various isothermal models such as Temkin, Henry, Frumkin, and Langmuir were used to investigate the surface adsorption isotherm present in the inhibited system. The Langmuir adsorption isotherm showed the best fit for the equilibrium adsorption data under investigation (see in Supporting Information Table S1). Equation 7 is used to draw the Langmuir isothermal model.^64^
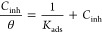
7where θ is the surface coverage inhibition efficiency (η) obtained from the PDP measurement  and *C*_inh_ and *K*_ads_ are the inhibition concentration and adsorption–desorption equilibrium constants, respectively. *K*_ads_ can be used to define the strength of the bond between the adsorbent and adsorbate. A correlation between *C*_inh_/θ and *C*_inh_ in Figure 11 resulted in a linear relationship with a regression coefficient (*R*^2^) of 0.99963. This indicates that the adsorption characteristics of the imidazoline molecules on the carbon steel surface closely follow the Langmuir adsorption isotherm model. As the slope of the line was close to 1, a reasonable correlation between the fitted results and experimental data was obtained. The *K*_ads_ value was 0.6058, calculated from the *y*-intercept of the regression line. The high *K*_ads_ value demonstrated a strong interaction between the inhibitor molecules and carbon steel surfaces and a high rate of inhibitor adsorption on the metal surfaces. Additionally, the *K*_ads_ value relates to the free energy of adsorption (Δ*G*_ads_^0^), given by the following expression (eq 8):
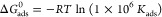
8where *R* (J/mol·K) is the ideal gas constant (8.3144598 J/mol·K), *T* (K) is the thermodynamic temperature, and 1 × 10^6^ (mg/L) is a constant that is correlated with the concentration of water molecules (at the same concentration as the inhibitor).^46^ The calculated Δ*G*_ads_^0^ value was −32.987 kJ/mol. In general, a Δ*G*_ads_^0^ value of −20 kJ/mol or less negative is associated with an electrostatic interaction or physisorption mechanism occurring between the inhibitor molecules and a charged metal surface. However, a Δ*G*_ads_^0^ value of about −40 kJ/mol or more negative is indicative of charge sharing or transfer from an organic species to a metal surface, which then forms a coordinate type of bond (referred to as a chemisorption mechanism).^41,46^ The adsorption mechanisms of S-Imd on a carbon steel surface therefore could be via both chemisorption and physisorption. This agrees well with several previous studies of imidazoline-type inhibitors.^18,35,48,50^

### Surface Morphological Studies

#### SEM and EDAX Analysis

**Figure 12:**
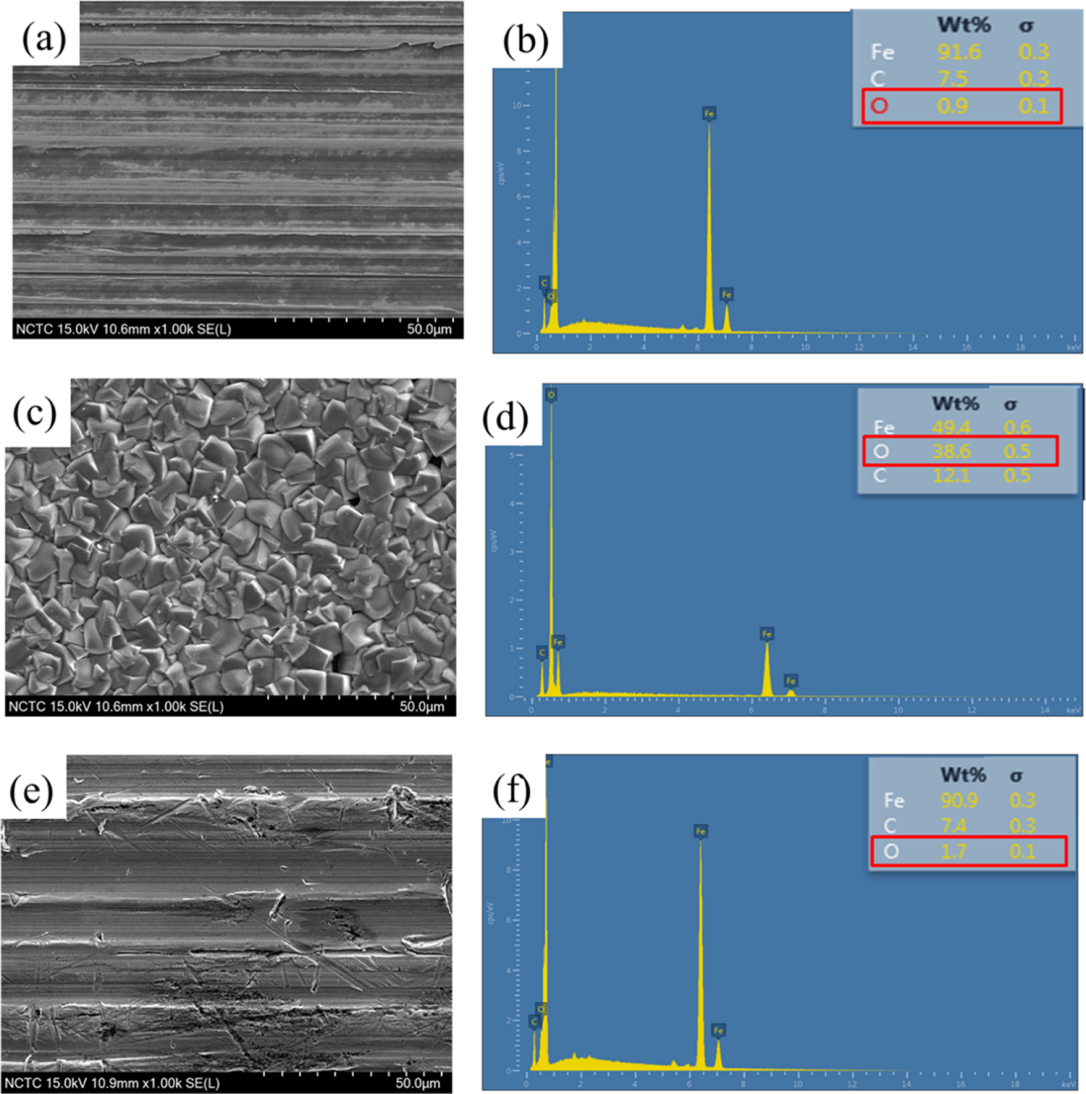
SEM images and EDAX mapping results on a carbon steel surface (a, b) in the polished state, (c, d) blank or uninhibited solution, and (e, f) inhibited system containing 300 ppm S-Imd.

SEM images and EDAX spectra were used to examine carbon steel surfaces in the polished state and after corrosion testing in a 1 wt % NaCl solution at 75 °C for 7 days under inhibited (containing 300 ppm S-Imd) and uninhibited conditions. In the absence of an inhibitor, Figure 12c,d reveals that the specimen surface is damaged with a higher oxygen level compared to the SEM image of a polished morphology and the corresponding EDAX mapping of Fe, C and O elements in Figure 12a,b. In an inhibited system containing 300 ppm S-Imd, smooth and fine polishing scratches reflecting light of the initial specimen surface could be clearly seen on the carbon steel surface after the TOL corrosion test in a 1 wt % NaCl solution. This observation confirmed the effectiveness of the adsorptive protective imidazoline film on the carbon steel surface. Formation of a protective film on the carbon steel surface was further examined using EDAX. The EDAX spectrum for the polished carbon steel (Figure 12b) shows the characteristic peaks of Fe, C, and O elements containing a high atomic percentage of Fe as the main constituent of the carbon steel. The atomic percentage of the Fe element on the metal surface decreased greatly, while the oxygen level was found to increase from 0.9 wt % (uncorroded specimen) to 38.6 wt % after the corrosion test under uninhibited test conditions (see Figure 12d). This indicates formation of an oxide layer on the carbon steel surface caused by the corrosion process. The test specimen subjected to inhibited conditions (Figure 12f) maintained its atomic percentage of Fe, C, and O elements at a level comparable to an uncorroded specimen. This result indicated that S-Imd was adsorbed on metal surfaces to form a protective film that prevented further dissolution of Fe through the formation of an oxide layer.

#### Contact Angle Analysis

**Figure 13:**
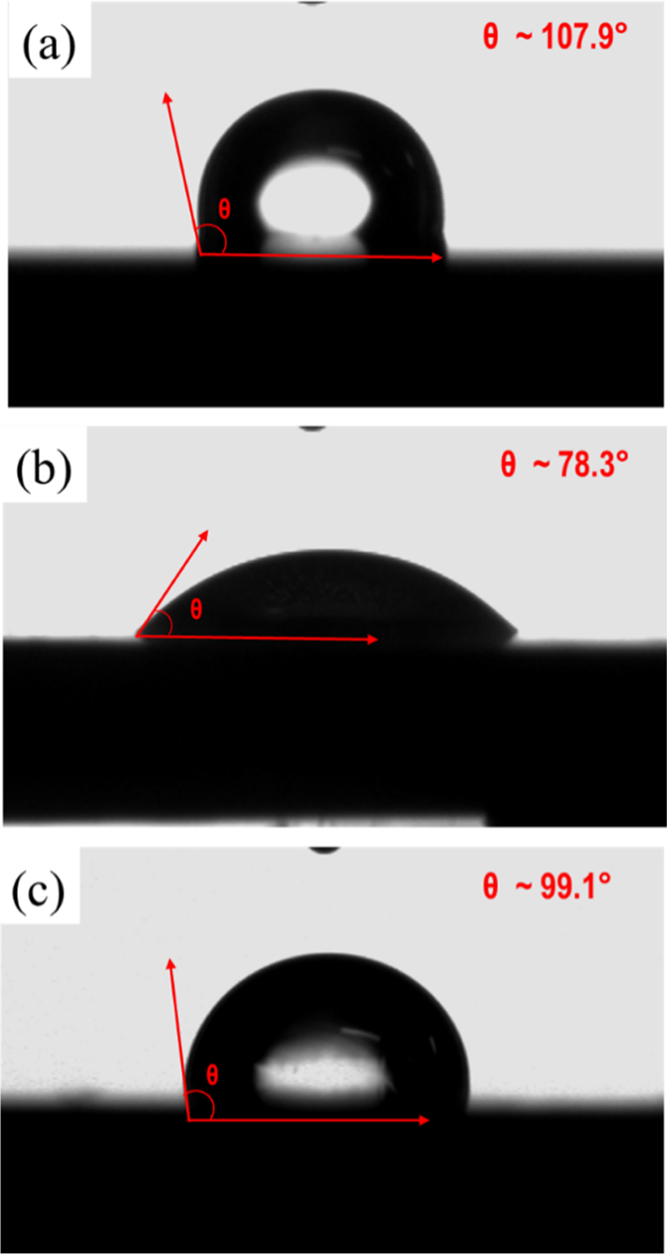
Contact angle on the carbon steel surface in (a) polished state, (b) blank solution, and (c) inhibited system containing 300 ppm S-Imd.

Figure 13 shows the results of a contact angle test on a carbon steel surface before and after TOL testing in 1 wt % NaCl with and without 300 ppm imidazoline inhibitor. The water contact angle for the uncorroded carbon steel surface was 107.9° (Figure 13a). After TOL testing in 1 wt % NaCl with no addition of inhibitor, the metal surface exhibited a decreased contact angle of 78.3° (Figure 13b) due to higher surface roughness under corrosive attack by the media. In contrast, the presence of a protective imidazoline film on the metal surface reduced the surface exposure to the corrosive media. Hence, there was little change in the surface roughness and the water contact angle (i.e., 99.1° as shown in Figure 13c). This is an indication of the hydrophobic character of the surface caused by adsorption of a protective imidazoline film that hindered water and oxygen from interacting with the steel surface. Similar observations were reported in previous studies.^7,18,46^ There was a significant correlation between the TOL weight loss measurements and surface analysis by contact angle analysis. For example, the carbon steel surface exposed to a blank solution had a lower contact angle (hydrophilicity) but a higher corrosion rate, whereas the opposite was seen under inhibited test conditions.

It is noted that future work on surface analysis is still needed to verify the effectiveness of S-Imd and to evaluate its action mechanism(s) as an efficient corrosion inhibitor by means of low energy electron diffraction (LEED), reflection high-energy electron diffraction (RHEED), Auger electron appearance spectroscopy (AEAPS), transmission electron microscopy (TEM), atomic force microscopy (AFM), etc.

## Conclusions

In this work, 2-(8-heptadecenyl)-2-imidazoline-1-ethanamin (S-Imd) was successfully synthesized via amidation and cyclization between S-TOFA and DETA with high yield. The evaluation of corrosion inhibition efficacy by electrochemical and weight loss measurements confirmed its suitability for use as a promising VCI agent for carbon steel pipelines (both at BOL and at TOL positions) in a CO_2_-containing environment. The inhibition efficiency increased with increasing inhibitor concentration. The dosage of 300 ppm reached maximum inhibition efficiencies of 91.6 and 89.9% in BOL and TOL corrosion tests, respectively. Electrochemical polarization results exhibited that the used inhibitors were mixed type but were predominantly governed by the anodic inhibitive effect (Δ*G*_ads_^0^ = −32.987 kJ/mol). The EIS studies indicated that the charge transfer resistance increased with increasing inhibitor concentration as a result of the adsorption of the inhibitor film on the metal surface according to the Langmuir adsorption isotherm. The physical characterizations using SEM, EDAX, and contact angle analysis confirmed that an effective protective layer of S-Imd molecules formed on the carbon steel surface.

## Supporting Information Available

Characterization of S-Imd and ^13^C NMR spectra, mass spectra, UV–visible spectra, adsorption isotherm, and corresponding equation of S-Imd (PDF)

## Abbreviations

BOL: bottom of the line
TOL: top of the line
EIS: electrochemical impedance spectrum
PDP: potentiodynamic polarization
LPR: linear polarization resistance
SEM: scanning electron microscopy
EDAX: energy dispersive X-ray spectroscopy
