# Supplementary material for: Imidazoline As a Volatile Corrosion Inhibitor for Mitigation of Top- and Bottom-of-the-Line CO2 Corrosion in Carbon Steel Pipelines
Source: Langmuir. 2024 May 30;40(23):11888–902. doi: 10.1021/acs.langmuir.3c03827 (PMC11171462; doi:10.1021/acs.langmuir.3c03827)
Supplement: Supplementary file 1 — la3c03827_si_001.pdf [file la3c03827_si_001.pdf]

Supporting Information

Imidazoline as a Volatile Corrosion Inhibitor for Mitigation of Top- and Bottom-of-the-Line CO2 Corrosion in Carbon Steel Pipelines

*Nattawut Yotapan [a], Nipaporn Sriplai [a], Sureeporn Ruengsangtongkul [a], Korakot Sombatmankhong [a,]**

National Energy Technology Center (ENTEC), National Science and Technology Development Agency (NSTDA), 114 Thailand Science Park, Phahonyothin Road, Khlong Nueng, Khlong Luang, Pathum Thani, 12120, Thailand

* Corresponding author: korakot.som@entec.or.th

Content

1. **Characterisation of S-Imd**

   1.1 Figure S1 ^{13}C NMR spectra of S-Imd 1

   1.2 Figure S2 Mass spectra of S-Imd 2

   1.3 Figure S3 UV-Vis spectra of S-Imd 3

2. **Adsorption isotherm**

   Table S1 Adsorption isotherm and corresponding equation 4

1. Characterisation of S-Imd (^{13}C-NMR, Mass spectrometer, UV-Visible spectrometer)

1.1 ^{13}C NMR spectra of S-Imd

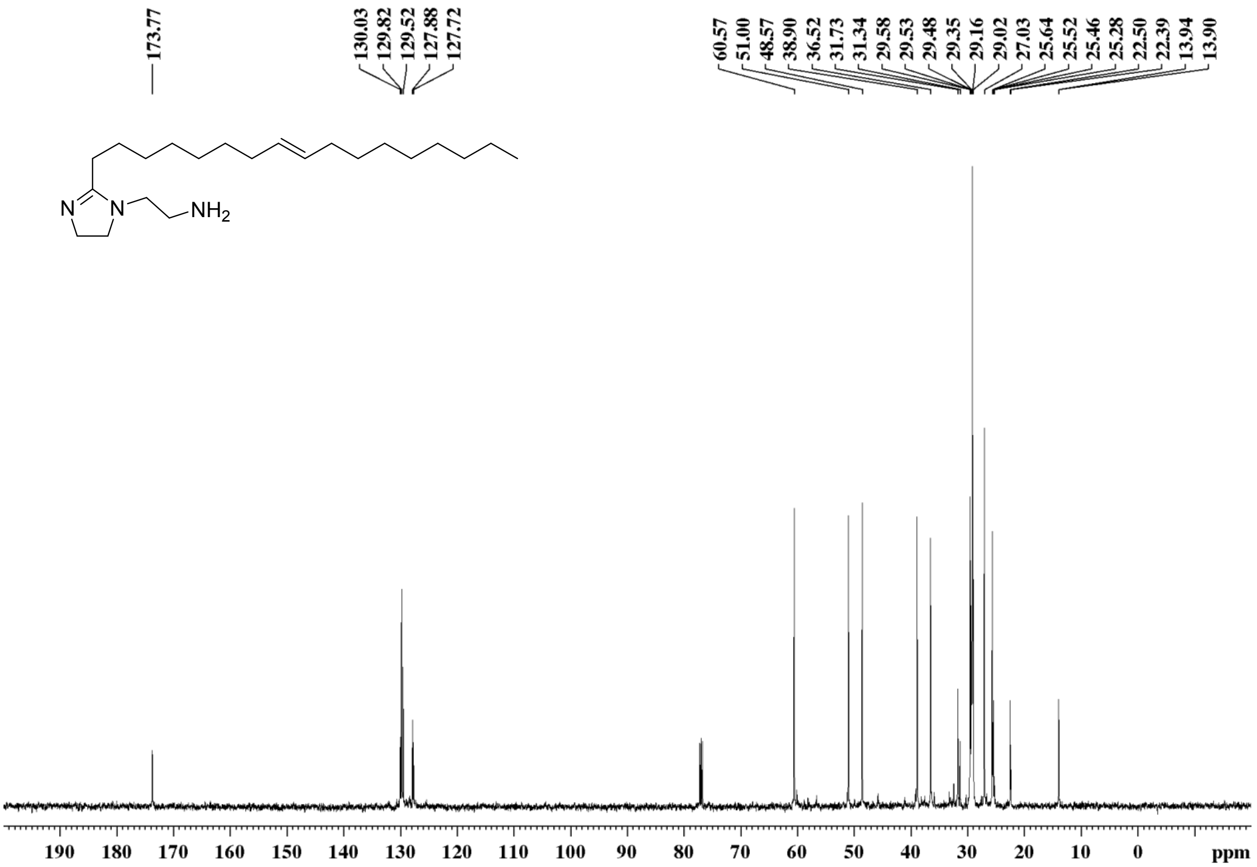

Figure S1 ^{13}C-NMR spectrum of S-Imd

1.2 Mass spectra of S-Imd

High resolution mass spectrometer of S-Imd

Mass (ESI) m/z : $(M)^+$ Calcd for $C_{22}H_{43}N_3$ 349.3457; Found 349.3203

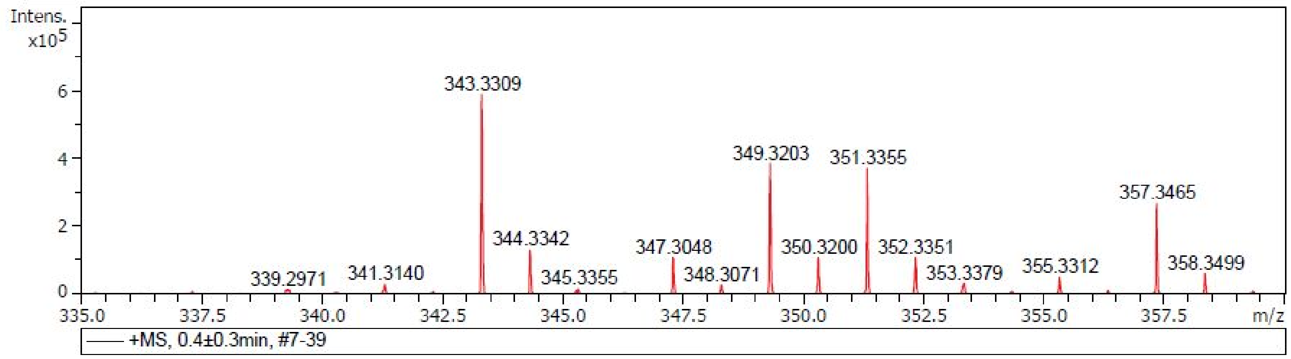

Figure S2 Mass spectra of S-Imd

1.3 UV-Vis spectra of S-Imd (λ_{max} = 260 nm)

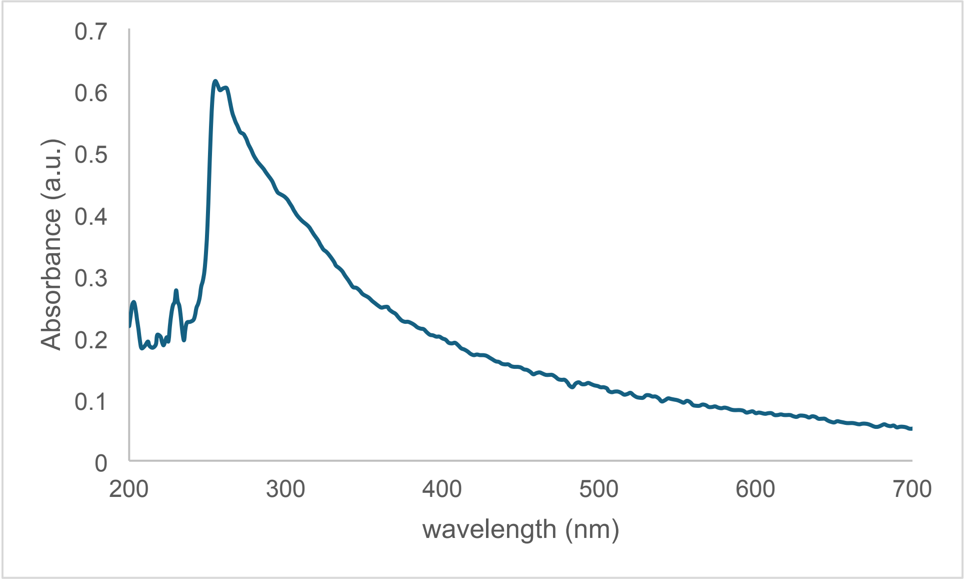

Figure S3 UV-Vis spectrum of S-Imd

2. **Adsorption isotherm**

Table S1 Absorption isotherms and the corresponding equation of S-Imd

Isotherm to be tested	Corresponding equation	R^2 (± 0.001)
Langmuir	$KC = \frac{\theta}{1} + \theta$	0.99963
Temkin	$KC = \exp(-g\theta)$	0.65343
Frumkin	$\theta = KC^n$	0.99714
Henry	$\theta = KC$	0.73677
